# A long-term travel delay measurement study based on multi-modal human mobility data

**DOI:** 10.1038/s41598-022-19394-z

**Published:** 2022-09-26

**Authors:** Zhihan Fang, Guang Wang, Yu Yang, Fan Zhang, Yang Wang, Desheng Zhang

**Affiliations:** 1 Department of Computer Science, Rutgers University, Piscataway, NJ 08854-8019 USA; 2 Department of Computer Science, Florida State University, Tallahassee, FL 32306 USA; 3 Department of Computer Science and Engineering, Lehigh University, Bethlehem, PA 18015 USA; 4 Shenzhen Institute of Advanced Technology, Chinese Academy of Science, Shenzhen, 518055 People’s Republic of China; 5 University of Science and Technology of China, No. 96, JinZhai Road, Hefei, 230026 Anhui People’s Republic of China

**Keywords:** Civil engineering, Sustainability

## Abstract

Understanding human mobility is of great significance for sustainable transportation planning. Long-term travel delay change is a key metric to measure human mobility evolution in cities. However, it is challenging to quantify the long-term travel delay because it happens in different modalities, e.g., subway, taxi, bus, and personal cars, with implicated coupling. More importantly, the data for long-term multi-modal delay modeling is challenging to obtain in practice. As a result, the existing travel delay measurements mainly focus on either single-modal system or short-term mobility patterns, which cannot reveal the long-term travel dynamics and the impact among multi-modal systems. In this paper, we perform a travel delay measurement study to quantify and understand long-term multi-modal travel delay. Our measurement study utilizes a 5-year dataset of 8 million residents from 2013 to 2017 including a subway system with 3 million daily passengers, a 15 thousand taxi system, a 10 thousand personal car system, and a 13 thousand bus system in the Chinese city Shenzhen. We share new observations as follows: (1) the aboveground system has a higher delay increase overall than that of the underground system but the increase of it is slow down; (2) the underground system infrastructure upgrades decreases the aboveground system travel delay increase in contrast to the increase the underground system travel delay caused by the aboveground system infrastructure upgrades; (3) the travel delays of the underground system decreases in the higher population region and during the peak hours.

## Introduction

We have more than 56.61% of the world population living in urban areas in 2021, and this number is projected to be 68% by 2050^1^, which leads to various mobility challenges, e.g., traffic congestion and energy consumption^2–4^. According to an analysis, drivers lost an average of 99 h due to congestion in 2019. The commuters in the New York City suffer from an annual delay of 102 h with a $1594.75 cost of congestion per driver in 2021^5^.

However, understanding long-term travel delay evolving patterns is extremely challenging because (i) travel delay happens in different modalities, e.g., subway, taxi, bus and personal cars, with implicated coupling, and (ii) long-term travel delay experiences spatial and temporal dynamics (e.g., city development, policy changes) under various contexts. More importantly, long-term multi-modal travel delay is difficult to model without explicated data.

Recently, the ubiquity of GPS devices and the upgrades of transportation infrastructures have led to unprecedented data for human mobility modeling including travel delay^6,7^. In particular, modern cities have been equipped with sensor devices in transportation systems to track and dispatch vehicles. Previous work has studied human mobility or travel delay based on real-world data such as cellphone data^8–14^, taxicabs^15^, buses^16^. These models have good performances when they are used to understand modal-specific mobility patterns and short-term travel delay, e.g., many papers using large-scale cellphone data^6,8^ to study daily commuting patterns. However, they cannot be used to understand long-term multi-model travel delay, e.g., cross-modality effect, since cellphone data do not have explicit transportation modality, e.g., subway, car, bus, and taxi.

In this paper, based on the smart city initiative of Chinese city Shenzhen^17^, we are working with Shenzhen Transportation Committee to access 5-year mobility data for major transportation modalities in both aboveground and underground transportation systems in Shenzhen for a data-driven transportation study. It enables us to analyze evolving mobility patterns and systematic evaluation in a long-term period for multiple modalities in a modern city Shenzhen with high-speed city development. Based on this data access opportunity, we conduct a measurement case study called mRhythm for urban travel delay measurement and understanding for Shenzhen long term transportation planning. To best of our knowledge, this is the first systematical data-driven measurement for travel delay based on large-scale, long-term, and cross-modality mobility data. mRhythm advanced the state-of-the-art works by providing several in-depth observations and causality analyses on long-term travel delay with the interaction of two major mobility modalities, i.e., aboveground systems including cars, taxis, and buses, and underground system including subways. In particular, we summarize our major findings in terms of 7 observations as follows:

- **Long Term Individual Modality Evolving** Based on our study from 2013 to 2017 of Shenzhen, we provide causality analyses for (1) one expected **Observation 1** the travel delay and variance increase each year for both aboveground systems (including cars, taxis, and buses) and underground system (including subways) (2) two unexpected observations: **Observation 2** The aboveground systems have a slow-down increase trend of travel delay compared to the underground systems, which means the rate of increase is higher for the underground systems than the aboveground systems; **Observation 3** The underground systems have a lower increased delay overall compared to the aboveground systems. More importantly, we provide in-depth analyses of 5-year census data including various factors, such as subway passengers, car numbers, population, length of subway systems and road networks, to explain our results.
- **Cross-Modality Impact Analyses** We made two new observations on how infrastructure upgrades of the underground system and the aboveground system can impact each other: **Observation 4** The aboveground system infrastructure upgrades (e.g., new highway) increase the underground system travel delay; **Observation 5** The underground system infrastructure upgrades (e.g., new subway lines) slows down the aboveground system travel delay increase. More importantly, we rigorously test the statistical significance of these observations and provide a case study for each of them to validate their representativeness.
- **Impact of Factors** We study the impact of five major contextual factors on travel delay including population, temporal, spatial, weather, and social event. We made two new observations. (1) Population: **Observation 6** The underground system travel delay decreases with higher population (from 15.5% delay in regions with 10K-20K population to 10.4% in regions with 50K–60K population); whereas the aboveground system travel delay increases with higher population (from 8% delay in regions with 10K–20K population to 28% in regions with 50K–60K population). (2) Temporal: **Observation 7** The underground system travel delay decreases during peak hours (from 18% delay in off-peak hours to 13% delay in peak hours); whereas the aboveground system travel delay increases during peak hours (from 35% delay in off-peak hours to 50% delay in peak hours).

## Results

**Figure 1:**
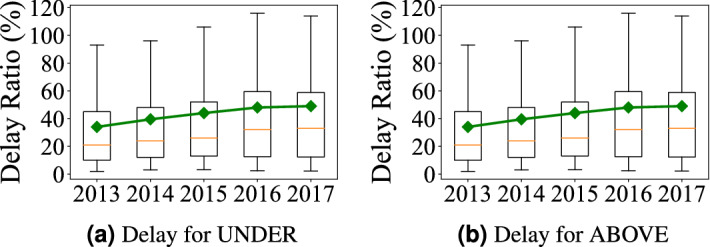
Travel delay from 2013 to 2017.

### Individual evolving patterns

#### Quantitative results

The box distribution of all trips’ delay is used where the top and bottom of each box are the 25th and 75th percentiles; the middle red lines are the median values; the top and bottom of each black dash lines indicate the maximal and minimal values. Figure 1a, b show the delay ratio distributions in underground systems (i.e., Subway) and aboveground systems (i.e., combining taxi, bus, and PV) from 2013 to 2017 where we made three observations as follows.

**Figure 2:**
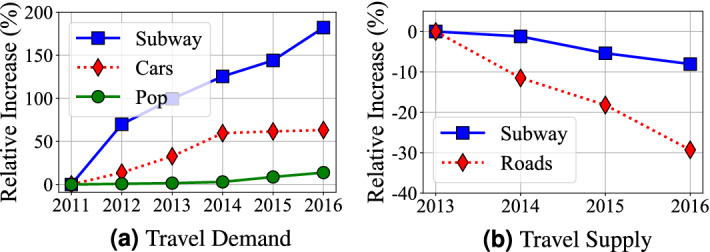
Travel demand and supply evolving.

*Observation 1* in Fig. 1: *For both underground and aboveground, both travel delay and variance increase each year* We analyze Shenzhen census data, which shows the high-level statistics about the mobility demand/supply and population change. We process the census data from 2011 to 2016 and normalize all absolute values to relative differences from the values of the first year, i.e., 2011, to show evolving patterns of travel demand and supply. *Increasing Overall Demand* Figure 2a gives Shenzhen Travel Demand by the number of passengers for subway; the number of personal vehicles (i.e., cars); Shenzhen citywide permanent population. We found that (1) the permanent population increases from 10.47 million to 11.91 million (13.8%); (2) the number of cars increases from 1.98 million to 3.23 million (63.3%); (3) the annual use of subway passengers increases from 0.4 billion to 1.2 billion (177.1%). *Decreasing per-capita Supply* Figure 2b shows Shenzhen Travel Supply by the length of roads per capita; the length of subway systems (in terms of km) per capita. We found that even increasing in the absolute length, both travel supply for both aboveground system and underground system decrease per capita due to increasing population and travel demand as shown in Fig. 2a. A combination of the increasing overall demand and decreasing per-capita supply leads to our observation.

*Observation 2* in Fig. 1: *The aboveground systems have a slow-down delay increase compared to the underground system* To explore the reason, we refer to Fig. 2a where we found that the increase of cars slows down from 2015. We further explore to find out that to control the increase of personal cars and alleviate the traffic congestion, from 2015, the Shenzhen government implemented a license lottery policy on personal cars^18^. In the license-plate lottery policy, residents submit applications for vehicle license plates each year but only certain number of applications will be approved. Therefore, the increasing speeds of personal cars became much slower since 2015. Since personal cars account for the major aboveground system delay, its slower car increase leads to our observation “the aboveground system has a slow-down delay increase”.

*Observation 3* in Fig. 1: *The underground system has lower increased delay overall compared to the aboveground one* To explore the reason, we refer to Fig. 2b where even both the underground system and aboveground system have decreasing per-capita supply, the underground system supply decreasing rate is much lower than that of aboveground system, due to the fast building of Shenzhen Subway System (i.e., increasing from 178.341 to 285.528 km in these 5 years). As a result, even though (1) the underground system has a strongly increasing demand (177.1%) and (2) the aboveground system has an almost-stopped increasing demand (due to license lottery policy, Shenzhen only have 2.2% more registered cars from 2014 to 2016), the underground system’s overall increase delay is still lower than the aboveground system. It might suggest that increasing supply is better than controlling demand.

#### Statistic significance of observation 1, 2, and 3

To validate the statistical significance of data for the above three observations, we put the average delay of all origin-destination pairs in a year in a vector, and then report the test results of the statistical difference between the delays of two consecutive years. Table 1 shows the results of two statistical tests, for each cell, the shadowed background means it is statistically significant in Mann–Whitney U test^19^ and the value in the cell is the ${\mathscr{A}}$ measure in Vargha–Delaney^20^. We found that all travel delay differences between consecutive years from 2013 to 2017 are statistically significant by both two tests. The ${{\mathscr{A}}}$ measure confirms the travel delay increases with time in the city, i.e., ${{\mathscr{A}}} > 0.5$. We observed a relatively smaller number in 16–17 when comparing ABOVE with UNDER, the potential reasons are increasing demand of UNDER, while the travel delay for ABOVE has a relatively small number due to car policy, a relatively larger baseline of delay for ABOVE in 2016, and upgrade of ABOVE road networks.

**Table 1:** Yearly travel delay difference.

Year	13–14	14–15	15–16	16–17
UNDER	0.71	0.74	0.76	0.79
ABOVE	0.72	0.75	0.78	0.71

### Cross-modal evolving patterns

For a rigorous cross-modal investigation, we study the impact of the aboveground system and the underground system infrastructure upgrades (e.g., new roads or new subway lines) on travel delay in two categories of areas: (1) Test areas (Test): city areas covered by the infrastructure upgrades based on our spatial partition. (2) Control areas (Control): city areas not covered. We calculate population distribution, delay ratio, and ratio increase speed in the test areas before infrastructure upgrades, and then we select control areas with similar features.

A travel delay ratio is defined with five attributes in Eq. 3 in both underground and aboveground systems. With the same $\theta$ and temporal partition, we apply a spatial alignment on the two systems to study the cross-modal impact under the same spatio-temporal dimension. Specifically, we map both the underground system and the aboveground system partitions into 497 administrative regions released by government based on the their overlap areas.

#### Impact of aboveground system infrastructure upgrades

We collect data about the aboveground system infrastructure upgrades (e.g., road open time) by analyzing OpenStreetMap data^21^ on social media. We found 653 km of roads were constructed and taken into services during 2013 and 2017, i.e., 6015 km of total road length at the beginning of 2013, and 6668 km of total road length at the end of 2017.

**Figure 3:**
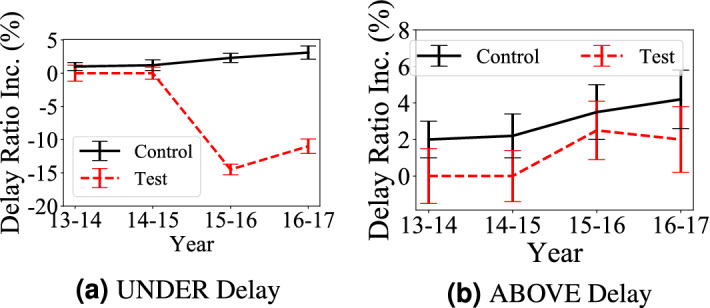
Delay increase in underground or aboveground areas covered by aboveground upgrades (test areas) and similar area not covered by aboveground upgrades (control areas).

**Citywide Trend** To compare the impact of aboveground system infrastructure upgrades (i.e., new roads) on travel delay in both aboveground and underground systems, Fig. 3 presents mean and standard deviation of delay ratio increase, i.e., the absolute delay ratio difference, during 5 years in the two groups.

*Observation 4* in Fig. 3a: *The aboveground system infrastructure upgrades increase the aboveground travel delay* When we compare test areas with control areas. Based on the locations of the aboveground system upgrade, we found that they mostly happen in suburban areas since the downtown areas are well-developed and out of space for new roads. As a result, these the aboveground system upgrades enable residents in suburban areas to take new roads to transfer to subways to go to downtown, which leads to increased the underground system travel delay in test areas. Based on results in Fig. 3b, we found some expected results: the aboveground system upgrades decrease the aboveground travel delay. This is an obvious observation since new roads decrease both travel distance in connected areas and travel volumes on existing roads.

**Table 2:** Impact of ABOVE upgrades.

Areas	Test before upgrade	Test after upgrade	Control before upgrade	Control after upgrade
UNDER	0.61	0.79	0.65	0.68
ABOVE	0.71	0.33	0.69	0.78

*Statistical Significance of Observation 4* We conduct U-test and A-test on different origin-destination pairs and the results are reported in Table 2. The shadowed cells indicate the travel delay differences are statistically significant with U-test. The number in the table is ${{\mathscr{A}}}$ measures of A-test. The results show the aboveground system upgrades have (1) a positive impact on the aboveground system delay, e.g., the aboveground system delay in Test Area decreases from 0.71 to 0.33 while the aboveground system delay in Control Area increases from 0.69 to 0.78; (2) a negative impact on the underground system delay, e.g., the underground system delay in Test Area significantly increases from 0.61 to 0.79 while the underground system delay in Control Area slightly increases from 0.65 to 0.68.

**A Case Study of Aboveground System Upgrades** We take a major highway (Guangshen Yanjiang Expressway) connecting Shenzhen airport, residential areas, and CBD areas for measurement, which was open at the end of 2013.

**Figure 4:**
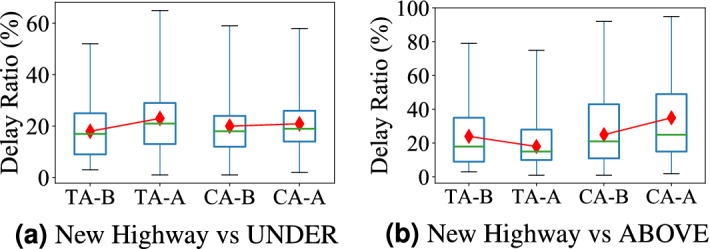
Impact of new highway; (**a**) new highway increases underground delay in test areas after open (TA-A); (**b**) new highway decreases aboveground delay in test areas after open.

Figure 4a shows the delay comparison for the underground system between the Test areas (impacted by the new highway) and Control areas (not impacted by the new highway) one month before and after this new highway was put into services. Similarly, Fig. 4b shows the travel delay comparison for the aboveground system.

(1) As shown in Fig. 4a, this highway significantly increases the underground system delay in the Test Area (from 18 to 24%) while the underground system delay in the Control Area only slightly increases (from 20 to 21%). This is because this highway (far-reaching to remote areas) brings more passengers for two major subway stations at one end of the highway, leading to increase the delay in these stations. This result is consistent with our Observation 4 on citywide trend. (2) As shown in Fig. 4b, this highway decreases the aboveground system delay in the Test Area (from 22 to 18%) while the aboveground system delay in the Control Area significantly increase (from 23 to 36%). The new highway directly decreases the travel time in the aboveground system by decreasing the travel distance and improving the traffic conditions. This result is consistent with our Observation 5 on citywide trend.

#### Impact of the underground infrastructure upgrades

In Shenzhen, three subway lines and 68 stations were added to the subway system in 2016 and 2017.

**Figure 5:**
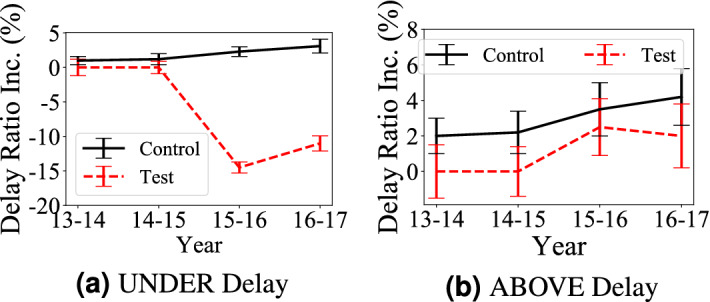
Delay increase in underground or aboveground areas with underground upgrades (test areas) and similar areas have no aboveground upgrades (control areas).

**Citywide Trend** To compare the impact of the underground system infrastructure upgrades (the underground system upgrade hereafter, i.e., new stations) on travel delay in both systems, Fig. 5 shows the delay ratio increase, i.e., the absolute delay ratio difference, during 5 years in the test areas (impacted by the underground system upgrades) and control areas (not impacted by the underground system upgrades but with similar demographic and geographic features). Since the aboveground system upgrade only happens in 2016 and 2017, the delay increases in 2014 and 2015 in test areas are 0.

*Observation 5* In Fig. 5a, an expected observation is that the underground system upgrades decrease travel delay in the underground system by 14% (absolute difference) due to increased underground supply. Figure 5b: *The aboveground system infrastructure upgrades slows down the aboveground system travel delay increase* From 3.5 to 2%, even though the travel delay still increases in test areas. We found most underground system upgrades happen in downtown areas with high travel demand. The underground system upgrades decrease travel demand in the existing aboveground system and lead to lower traffic increase in the aboveground system. This is also supported by the previous literature outcomes such as a mismatch between demand increase and travel infrastructure upgrades^22–24^.

*Statistical Significance of Observation 5* We further measure the difference by the two tests, and their results are reported in Table 3. The shadowed cells show the travel delays are statistically significant by U-test. The values of ${{\mathscr{A}}}$ measure show underground upgrades have (i) a positive impact on the aboveground travel delay, e.g., the ${{\mathscr{A}}}$ measure of aboveground travel delay in Test Areas slows down from 0.68 to 0.53 while the ${\mathscr{A}}$ measure of aboveground travel delay in Control Areas increases from 0.66 to 0.71; (2) a positive impact on the underground travel delay, e.g., the ${\mathscr{A}}$ measure of underground travel delay in Test Areas decreases from 0.61 to 0.28 while the ${{\mathscr{A}}}$ measure of underground travel delay in Control Areas increases from 0.61 to 0.63.

**Table 3:** Impact of UNDER upgrades.

Areas	Test before upgrade	Test after upgrade	Control before upgrade	Control after upgrade
UNDER	0.61	0.28	0.61	0.63
ABOVE	0.68	0.53	0.66	0.71

**Figure 6:**
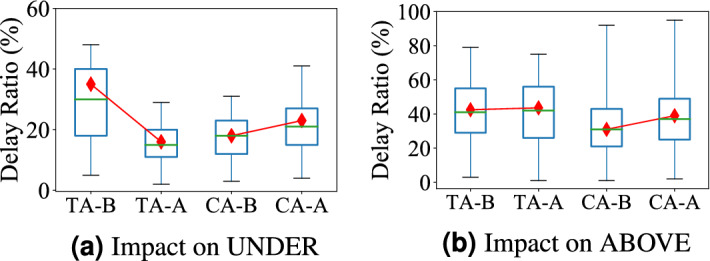
Impact of new stations; (**a**) it decreases the travel delay in the underground system; (**b**) it decreases the travel delay in the aboveground system.

**A Case Study of Underground System Upgrades** We investigate the impact of subway line NO.7 opened in 2016, which has a length of 30.1 km and includes 27 stations. For the underground system, we study how the new stations influence travel delays of overloaded stations. Figure 6a shows the travel delay comparison for the underground system. Figure 6b shows the travel delay comparison for the aboveground system between Test areas (impacted by the new subway line) and Control areas (not impacted by the new subway line) one month before and after this new subway line was taken into services.

(1) As shown in Fig. 6a, this new subway line decreases underground delay in Test areas significantly (from 36 to 18%) while the underground system delay in control areas increases slightly (from 19 to 20.6%). This is because the subway line passes through central business areas and is connected with 6 existing subway lines. It reduces passengers in existing stations, reduce the travel distance and transfer time. The result is consistent with our Observation 6 on citywide trend. (2) As shown in Fig. 6b, the new subway line slows down the travel delay increase in the Test areas (0.9% delay ratio increase compared with 3.2% delay ratio increase in Control areas). This is because more passengers choose the subway and it slows down the passenger demand increase in the aboveground systems. The result is consistent with our Observation 5 on the citywide trend.

### Contextual factors

We measure the impact of five contextual factors on delay ratio of the underground system (i.e., subway) and the aboveground system (i.e., car, bus, taxi), i.e., population, temporal, spatial, weather, social event, and West Texas Intermediate (WTI) crude oil price.

**Figure 7:**
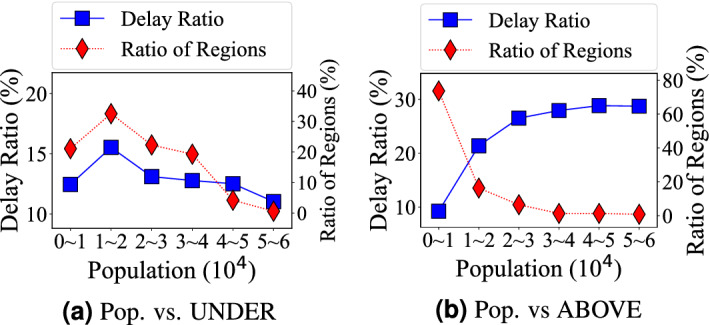
Correlation between travel delay and population: (**a**) the underground system’s delay is low in populous regions; (**b**) the aboveground system’s delay is high in populous regions.

**(1) Population**

Population distribution is one of the most important and unexpected factors for travel delay. For the underground system, we map the population to stations based on the walking distance in a non-overlapping partition where the average distance between adjacent stations in the subway system is 1.16 km, so we set the walking distance as half of the average distance, i.e., 0.58 km, which leads to the walking area of $1.05\,{\text {km}}^2$^25^. 78.4% of regions in the coverage areas of the subway system have more than 10,000 population because the subway system covers the most downtown areas. For the aboveground system, we map the population to grids and investigate the relationship between population and delay at the grid level. The grid size we investigate is $1.37\,{\text {km}}^2$. 77.1% of grids have less than 10,000 population. In Fig. 7a, b based on different populations on the X-axis, we show both the ratio of regions among all regions (Left Y-Axis) and average the underground system delay in these regions (Right Y-axis). *Observation 6* the underground travel delay decreases with the higher population; whereas the aboveground system travel delay increases with the higher population. This is mainly because of the different flexibility of underground system and the aboveground system supply, even with the same increasing demand. For the underground system in high population regions, both higher train frequency and denser subway system deployment are used for higher the underground system supply per passenger in populous areas. However, for the aboveground system, a higher the aboveground system supply is not possible in populous regions since the space for the aboveground system infrastructure upgrades (e.g., new road) is limited in the populous regions. Another minor observation is that the degree of the aboveground system travel delay increasing is smaller in grids with more than 20,000 population. This may be because the dense regions (e.g., downtown CBD) have better road structures.

**Figure 8:**
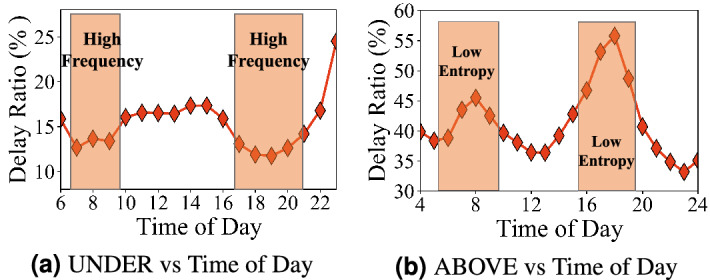
Delay distribution during one day; (**a**) underground travel delay is dominated by train frequency; (**b**) aboveground travel delay is dominated by spatial entropy.

**Figure 9:**
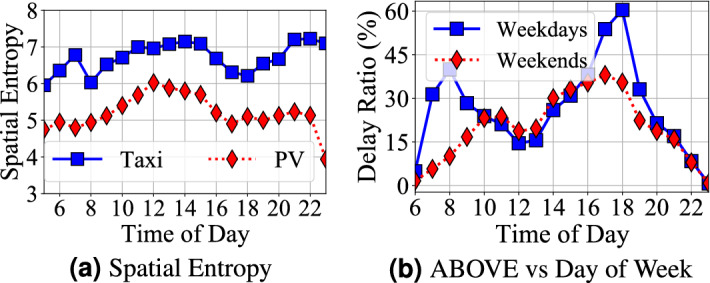
(**a**) A lower spatial entropy means lower randomness on spatial dimension and cars moves to certain areas; (**b**) lower peak hour and shifts on weekends.

**(2) Temporal** Fig. 8 presents travel delay during one day in UNDER and ABOVE system. *Observation 7* the underground system travel delay decreases during peak hours (7 am to 9 am and 4 pm to 8 pm); whereas the aboveground system travel delay increase during peak hours. For example, there are a lower travel delay in the underground system (around 13% on average) and a higher travel delay in the aboveground system (around 50% on average). For the underground system, the subway system sends trains more frequently in peak hours as shown in Fig. 8a, i.e., the 5-min time interval in the peak hours and the 8.5-min time interval in the off-peak hours^26^. Sending trains more frequently reduces the waiting time significantly while increases the operational cost. For instance, a subway line with 1 h of total travel time requires at least 7 subway trains (1 h/8.5 min) to maintain services in off-peak hours but 12 trains in peak hours (1 h/5 min). It increases the operational cost by 71% (from 7 trains to 12 trains). For the aboveground system, we found a lower entropy (lower randomness) in taxis and personal vehicles as shown in Fig. 9a during the peak hours. In other words, taxis and personal cars concentrate on commuting between certain areas, e.g., work areas and home areas, during peak hours, which leads to increased the aboveground system delay. Figure 9b shows the weekly patterns where we found a higher travel delay on weekdays than weekends in the aboveground system by 25% during peak hours, while the difference is negligible in the underground system.

**(3) Spatial** In both systems, travel delay increase by years in downtown areas because of higher travel demand in the downtown areas (where most regions with Top *k* delay ratios reside) with a limited improvement of infrastructures. In contrast, the general trend of suburban areas in both systems is decreasing by years. This is because the better infrastructures are constructed in suburban areas, e.g., roads and subway stations, along with Shenzhen urbanization. Comparing the underground and aboveground systems, we found the impact of infrastructure upgrades on the underground system is larger than the aboveground system because a new subway line can cover large areas and can even function as transfer lines to decrease the travel time between the existing two stations.

**Figure 10:**
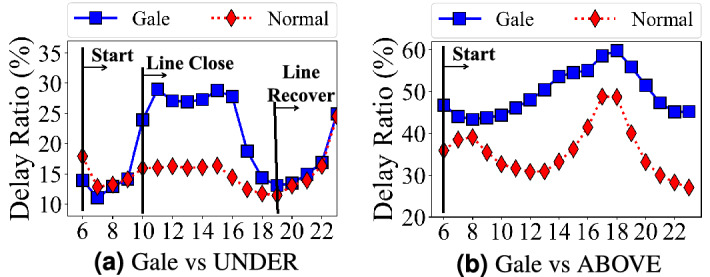
Extreme weather causes high travel delay in both underground and aboveground system; caused (**a**) subway lines close and (**b**) low speed on roads.

**(4) Weather** We group days in 5 years by the level of wind (fresh, gentle, moderate, gale), rain (no rain, light rain, heavy rain), and temperature.

*(i) Wind* Figure 10a, b show the delay changes in time of day under different wind levels. For the extreme wind weather (e.g, gale), both aboveground system and underground system have higher delay ratios. Through a deeper analysis, we found the delay in the underground system is caused by the temporary closing of some lines and stations where passengers take detours, which leads to higher the underground system delays. But even though some stations and lines may be closed in extreme weather, the underground systems might be more reliable since it can be recovered soon. *(ii) Rain* For the aboveground system, both the light and heavy rains increase travel delay, especially during peak hours. For the underground system, the light rain increases the travel delay; whereas the heavy rain slightly decreases the travel delay, which is caused by the decrease of passenger demand after analyzing the number of passengers in the subway system. *(iii) Temperature* Through our analyses, there is no major impact of temperature on travel delay.

**(5) Social Events** We found different social events have different impacts on travel delay. For example, in the Spring Festival, since most residents leave the city, the travel delay decreases a lot during that time; whereas in the National Day Break, people go shopping and go to local attractions, which leads to a high travel delay.

**Figure 11:**
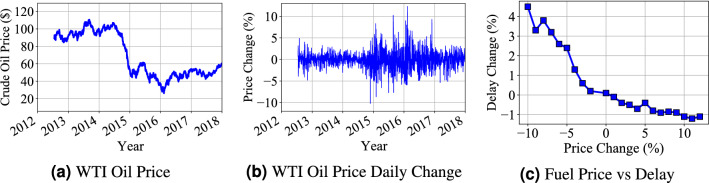
Fuel price change causes the dynamics of delay ratio for above-ground transportation.

**(6) Crude Oil Prices** Figure 11a gives the WTI crucial oil price changes based on the public data records^27^. The general trend of crucial oil prices decreased from around 100 USD to around 50 USD during year 2012 to years 2018. The correlation analysis is based on the WTI crucial oil price and travel delay ratio change. Since the delay ratio increases in long term due to city expansion and increasing demand, we conduct impact analysis of oil price change in a short-term period. Figure 11b shows the WTI crucial oil price changes on daily basis (relative difference of day two compared with day one). We compared the delay ratio change with the fuel price change in Fig. 11c, in which we found a negative correlation between delay ratio change and fuel price changes. The decrease of fuel price has a higher impact on delay ratio compared with the increase of the price. The potential reason is that the decrease of fuel price will increase the travel demand on above-ground transportation while the increasing price does not impact the travel demand of people who already choose to drive cars to commute. Besides, the impact of fuel price in short term does not impact underground transportation significantly because it does not change the train frequency in the underground transportation system.

## Discussion

**(i) Delay Evolving** Based on our long-term measurement, we provide some qualitative and quantitative results (Fig. 1a, b) to show delay increasing for both systems. Our measurements show the travel increases are statistically significant and the significance increases by years (Table 1). The main reason for delay increasing given by our causality analyses is a long-term mismatch between demand increase and travel infrastructure upgrades (Fig. 2b).

**(ii) Contexts**

The most unexpected results are the impacts of population on these two systems. For the aboveground system, a region with higher population has a higher delay (Fig. 7b); whereas for the underground system, a region with higher population has a lower delay (Fig. 7a). We found the fundamental reason for this contradiction is the transportation resources per capita for these two systems. Social events either increases or decreases delay based on resultant culture-based patterns.

**(iii) Interdependency** We found that delay dependency exists between different modalities. In the underground system, having new subway lines and stations decreases the delay by 44.1% in the overloaded transfer stations, and remits the travel delay increases in the aboveground system from 43.8 to 13% (Fig. 6b, a). In the aboveground system, having new highway decreases the travel delay in the connected regions by 12.2% (Fig. 4b), but it increases the travel delay in the connected subway stations by 16.7% because it connects subway uncovered areas with subway stations, leading to high subway demand (Fig. 4a).

**(iv) Practical Impacts on Shenzhen** Our measurement results and observations have practical impacts on Shenzhen Transportation Committee. The long-term individual modality and its causality analysis provide some comprehensive and quantitative references for their evolving patterns in Shenzhen covering both aboveground and underground mobility patterns. Our cross-modality impact analysis gives a new angle for mobility analysis in Shenzhen because most of existing studies are based on single modalities, and ignore the impacts from other transportation modalities, which may lead to bias in modeling. We studied various contextual factors in mobility modeling and analyses, but most of existing works are focusing on short-term and single modality instead of long-term analysis with cross-modality settings. Those are all important factors for researchers’ and practitioners’ projects focusing on Shenzhen human mobility modeling, traffic volume and speed predictions, urban planning. For working with Shenzhen Transportation Committee, we have achieved our goals to understand transportation infrastructure deployment (e.g., select best locations for new subway stations) and public transportation scheduling (e.g., frequency of subway and bus lines) from a long-term and multi-modal perspective. Our study indicates that the underground infrastructure upgrades (e.g., new subway lines or stations) might decrease both the travel delay in the underground system and aboveground system, e.g., a new subway line in Shenzhen reduced underground delay by 18% and reduced underground delay. Hence, new subway stations or subway lines need to be built to reduce travel delay.

**(v) Broader Impacts on Other Applications and Data Release** Human mobility is a key topic of Data Science Technology, and one of the most important measurement to human mobility evolution is the travel delay change in a long time. We investigate the first long-term multi-modal travel delay evolution at city scale, which potentially reflect the general human mobility trend. Our insights and lessons learned can benefit a wide range of future urban mobility applications, i.e., multi-modal travel recommendation^28,29^, congestion-aware fleet management^30^, social event detection^31^, human mobility prediction^32,33^, measuring the impact of mobility intervention strategies for the recent pandemic mitigation^6,34^. The one month aggregate multi-modal data set we will release has the potential to motivate and validate the aboveground urban mobility solutions, and verify various human mobility models for broader applications.

**(vi) Generalization** Despite the fact that our measurement work is done in one city, i.e., Shenzhen, the measurement framework such as metrics and insights can be potentially generalize to other urban cities with similar scales and policies, e.g., related to the observation 2 and 3. However, most of our observation is independent from local policies, which we believe can be generalized to other cities as well. Specifically, we can also measure the impacts of contextual factors on aboveground and underground travel delay given the similar data from any particular city.

**(vii) Limitations** We only use data from a particular city Shenzhen for a travel time delay measurement, so the results and implication may only apply to Shenzhen or cities with similar features. But we believe our results can provide new insights for urban planners or transportation developers to improve mobility of cities with similar features. We study the travel delay based on a prefixed spatial partition, which may be not the optimal partition for delay measurement, but how to partition cities into different regions to understand human mobility are still an open question^35^. Due to limited data access, we only consider the aboveground motorized modalities, e.g., taxi and personal cars, and other non-motorized modalities, e.g., biking or walking, will also provide new insights for travel delay modeling. Another limitation of this work is the time duration of the data, which is from 2013 to 2017. Prolonging the time series and reflecting more recent data in the analyses would improve the paper. However, in practice, it is challenging for researchers to obtain such a long-term large-scale dataset from four different transportation systems. In this work, we are working with the Shenzhen government, who provides the data between 2013 to 2017 to us for its urban sustainable developments. Since 2017, there are stricter data access regulations in China , it is hard to obtain such large-scale human mobility data after 2017. Even though the data are 5 years old, we argue that our long-term measurement study can still reveal travel dynamics and the impact among multi-modal systems during the evolving process with different factors like infrastructure upgrading, population growing, and weather change, etc. Since this is also the first work to investigate the long-term travel delay change with 5-year dataset collected from four transportation systems with 8 million residents, we believe the findings in this paper can benefit other researchers and city governments. In addition, the measurement framework can be potentially generalize to other urban cities with similar scales and policies since most of our observation is independent from local policies.

**(viii) Modal Shifting During the Pandemic Situation** During the COVID-19 pandemic^36,37^, there is an increase in the usage of personal cars instead of public transport in some cities^38,39^, which may cause more congestion and travel delay of the aboveground system^39,40^. One way to relieve this situation is to increase the punctuality of public transportation systems (e.g., providing exclusive bus lanes), which would be useful to gain back the trust of those passengers who shifted to cars during the pandemic. In addition, more residents choose to work remotely for safety considerations, which can also restrict the number of used private cars and increased delay time during rush hours.

## Methods

### Multi-modal dataset and statistics

**City Background** We accessed both the system data and contextual data from Shenzhen. As one of the most modernized cities in China (one out of four tier-1 cities with rapid urbanization), Shenzhen is located in the southeast seashore of China with a size of 792 $mi^2$ and 12 million population.

**Figure 12:**
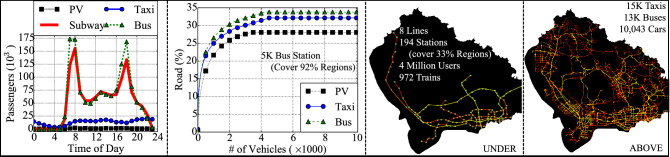
Mobility dataset in Shenzhen.

**Mobility Data** We utilize four different urban transportation systems in Shenzhen, i.e., subway, bus, taxi, personal car. The data cover all of the city population who utilize smartcard for public transportation (bus and subway), all of the passengers in the city who take taxis, and 10 thousand personal car drivers. (i) *Subway System* includes 8 lines, 194 stations, 972 trains, 3 million daily passengers, and 4 million daily fare records. Passengers swipe smart cards when they enter origin stations or leave destination stations. A fare transaction record has 6 attributes (*card id, date, time, station id, in/out*). (ii) *Bus System* includes 1115 bus lines, 10,106 bus stations, and 13 thousand buses. A bus record (uploaded every 30 s) has 6 attributes (*plate, date, time, stop id, GPS location, speed*). (iii) *Taxi System* includes 15 thousand taxis. A taxi record (uploaded every 30 s) has 6 attributes (*plate, date, time, GPS, speed, free/occupied*). (iv) *Personal Vehicle System* (i.e., PV system) includes 10 thousand cars traveling regularly around Shenzhen. A PV record (uploaded every 10 s) has 5 attributes (*device id, date, time, GPS location, speed*). Drivers voluntarily report their PV data with an on-board diagnostics and a smartphone app for insurance premium discounts.

**Figure 13:**
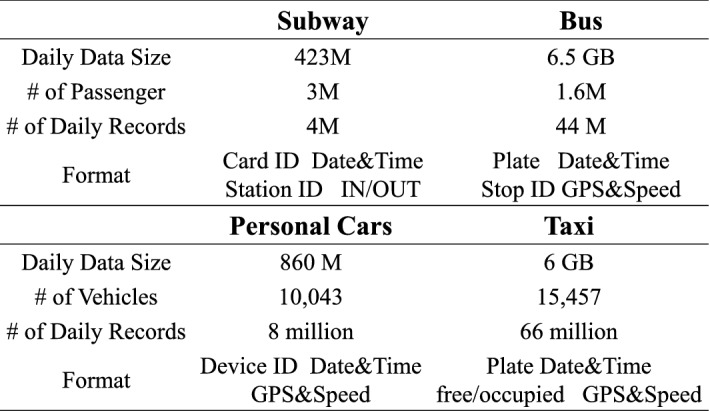
Mobility dataset.

Figure 12 is drawn by us using a software called Processing version 3.5.4^41^. The first two figures in Fig. 12 present temporal demand distribution, i.e., number of passengers, of the four kinds of mobility, assuming one passenger per PV, and spatial coverage of three aboveground mobility. They are rather expected. The last two figures in Fig. 12 visualize the four mobility with two categories, i.e., the underground system (i.e., subways) and the aboveground systems (i.e., buses, taxis, cars). The brighter color indicates higher travel demand. For example, the underground system covers some populous areas in a city, e.g., downtown areas; whereas aboveground system cover the majority of the city. We omit further statistics since they are expected. A summary of the mobility dataset is shown in Fig. 13.

### Partition and metrics

**Figure 14:**
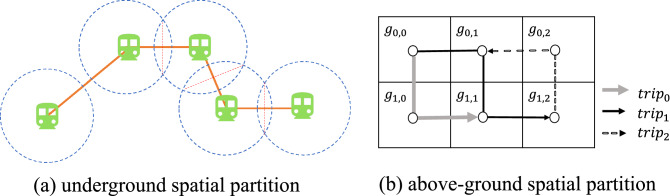
Spatial partition.

*Spatial Partition* We use different spatial partitions in (1) the aboveground systems including taxis, buses, personal cars; (2) the underground system including subway. For aboveground systems, the most delays are due to traffic congestion on roads^25^; whereas for the underground system, most of the delay is due to the waiting in the subway station^42^. Moreover, a underground trip has fixed stations as origins and destinations (OD hereafter); whereas an aboveground trip has flexible OD at arbitrary locations given by GPS. Figure 14a presents a partition for the underground system based on subway station coverage where we apply a non-overlapping partition with walking distance circles and perpendicular bisector lines to assigning residents to the nearest station within walking distance. Figure 14b presents a grid partition for the aboveground systems with an example of three trips. To ensure fine-grained modeling, we divide the Shenzhen into $100\,{\text {m}} \times 100\,{\text {m}}$ rectangular grids, which is used extensively for fine-grained mobility modeling^25^.

*Trip Travel Time* Given the above aboveground system spatial partition, we first apply an existing trip segmentation technique based on staying points^43^ to extract the aboveground systems trips (Note that the underground system trips are directly given by data). We identify a trip by five parameters $(s_o,s_d,t_o,t_d,i)$, where ${s_o}$ and ${s_d}$ are the origin and destination of the trip, ${t_o}$ is the start time of the trip, ${t_d}$ is the end time of the trip, *i* is the *passenger id* or *vehicle id* of this trip. Given a trip, its travel time is the time difference between the trip end time and trip start time, which is given by a function ${\tau }(s_o,s_d,t_o,t_d,i)$. The travel time of a trip is given in Eq. (1).

1 $$\begin{aligned} {\tau }(s_o,s_d,t_o,t_d,i) = t_d - t_o. \end{aligned}$$

*Fluent Travel Time* When no congestion occurs in a trip between an OD pair, the travel time of this trip is called the fluent travel time of this OD pair, which is often modeled as the minimum travel time between two locations^25^. However, since lots of errors and noise exist in real-world data caused by factors such as sensing errors or abnormal behaviors (e.g., motor racing), the minimum travel time is most likely to be an error. Therefore, we first apply a data clean procedure, and then we use a noise filer function to remove potential errors and noise^43^. Therefore, the fluent travel time estimation is given in Eq. (2) where we filter errors and noise by a predefined threshold of the standard score x (i.e., z score) $\theta$ on travel time $\tau$.

2 $$\begin{aligned} \begin{aligned}{}&{f(s_o,s_d,\theta )} = \min _{i,t_o,t_d} \{{\tau (s_o,s_d,t_o,t_d,i)}| P(\tau \leqslant z) \leqslant \theta \} \\&z = \frac{|{\tau (s_o,s_d,t_o,t_d,i) - \bar{\tau }(s_o,s_d,\forall t_o, \forall t_d, \forall i})|}{\sigma ({s_o,s_d, \forall t_o, \forall t_d, \forall i})} \end{aligned} \end{aligned}$$

*Delay Ratio as Measurement Metric* Based on a trip *i*’s travel time $\tau (s_o,s_d,t_o,t_d,i)$ and the fluent travel time $f(s_o,s_d,\theta )$ between two locations $(s_o,s_d)$, the **Delay Ratio** of this trip *i* is defined as in Eq. (3).

3 $$\begin{aligned} \begin{aligned} {d(s_o,s_d,t_o,t_d,i,\theta ) = \frac{\tau (s_o,s_d,t_o,t_d,i)-f(s_o,s_d,\theta )}{f(s_o,s_d,\theta )}} \times 100\% \end{aligned} \end{aligned}$$

We set $\theta =1.96$ which gives us a 95% confidence interval. We use subway fare records to model travel delay ratio of the underground system, and on-board GPS records to model the travel delay ratio for above-ground systems, Even though the data format are different, we use the same method to model the delay ratio. In the first step, we calculate the travel time for individual trips between an origin-destination pair by Eq. 1. Second, we model the fluent travel time between origins and destinations with Eq. 2. In the last step, we use Eq. 3 to calculate the delay ratio of individual trips.

*Statistical Significance of Delay Ratio Comparison* To ensure a rigorous delay ratio study beyond traditional metrics (e.g., standard deviation), we apply the non-parametric significant test, i.e., Mann–Whitney U test^19^, to determine if a difference between travel delays are statistically significant (*p* value below 0.01) by chance. To further investigate how much two sets of travel delay differs, we use a non-parametric effect size, a pair-wise Vargha-Delaney A measure^20^ as in Eq. (4).

4 $$ \begin{aligned}   {\mathcal{A}}(D_{i} ,D_{j} ) &  = \frac{{\sum\limits_{{k = 1}}^{n} \sigma  (d_{k}^{j}  - d_{k}^{i} )}}{n} \\    \sigma (d_{k}^{j}  - d_{k}^{i} ) &  = \left\{ {\begin{array}{*{20}l}    {1,\;\;d_{k}^{j}  - d_{k}^{i} } \hfill & { > 0} \hfill  \\    {0.5,\;\;d_{k}^{j}  - d_{k}^{i}  = 0} \hfill & {} \hfill  \\    {0,\;\;d_{k}^{j}  - d_{k}^{i} } \hfill & { < 0} \hfill  \\   \end{array} } \right. \\  \end{aligned}  $$

where *D* is a set of travel delays, e.g., ${D}_i = \{d_1^i,\ldots ,d_k^i,\ldots ,d_n^i\}$ and ${D}_j = \{d_1^j,\ldots ,d_k^j,\ldots ,d_n^j\}$. For example, ${D}_{2013}$ is the travel delay of the Year 2013 between all origin-destination pairs; $d_k^{2013}$ is the average delay between one OD pair *k*. The pair-wise Vargha-Delaney A measure (${\mathscr{A}}$ measure hereafter) indicates the probability that one travel delay distribution is higher than another. ${\mathscr{A}}$ measure is above 0.5, the first distribution is higher; ${\mathscr{A}}$ measure is 0.5, the two distributions are equal; ${\mathscr{A}}$ measure is below 0.5, the second distribution is higher. The closer ${\mathscr{A}}$ measure to 0 or 1.0; the higher the differences between the two distribution.

In this work, we only utilize the aggregate data to study the travel delay, and the individual information cannot be revealed from our data. Any sensitive information has been removed before conducting this project. We confirm that informed consent was obtained from all subjects and/or their legal guardian(s). For example, before the data were collected, all passengers and drivers have been notified that their transportation card data or commercial vehicle GPS data will be collected for the billing and mobility systems management purposes including understanding mobility system performance and improving mobility services. We confirmed that all methods were performed in accordance with relevant guidelines and regulations. We confirmed that all experimental protocols were approved by the Institutional Review Board of School of Software Engineering, University of Science and Technology of China.

### Conclusion

In this paper, we quantify, measure, and understand long-term multi-modal travel delay based on a 5-year multi-modal mobility dataset in Chinese city Shenzhen. Our measurement study utilizes a dataset of 8 million residents from 4 transportation modalities spanning 5 years from 2013 to 2017, which, to our knowledge, has not been studied before due to limited data access. Through our measurement, we found: (1) the aboveground system has a higher delay increase overall than that of the underground system but the increase of it is slow down; (2) the underground system infrastructure upgrades decreases the aboveground system travel delay increase in contrast to the increase the underground system travel delay caused by the aboveground system infrastructure upgrades; (3) the travel delays of the underground system decreases in the higher population region and during the peak hours.

## Supplementary Information

Supplementary Information 1.

## Data Availability

Codes to reproduce our results in the figures from the data is publicly available on github. https://github.com/GrandDuelist/mRythm.
